# Effect of social cognitive mindfulness on preschool teachers’ professional identity: the mediating role of resilience

**DOI:** 10.3389/fpsyg.2025.1587965

**Published:** 2025-10-13

**Authors:** Yanmei Tang, Ziyue Wang, Zongjin Yuan

**Affiliations:** ^1^School of Early-Childhood Education, Nanjing Xiaozhuang University, Nanjing, China; ^2^Guanyun County Longju Central Primary School, Lian Yun Gang, China

**Keywords:** preschool teachers, mindfulness, professional identity, resilience, implicit association test

## Abstract

The current study investigates the effect of social cognitive mindfulness (SCM) on professional identity and the potential mediating role of resilience in this relationship. The professional identity of preschool teachers is assessed both explicitly using a self-reported scale and implicitly using the Implicit Association Test [SC-IAT]. A total of 106 Chinese preschool teachers were randomly recruited from Jiangsu Province. Participants completed the SC-IAT and several questionnaires. Results show that SCM significantly improved the preschool teachers’ professional identity, both implicitly and explicitly. Results also reveal that resilience plays a mediating role in the relationship between SCM and professional identity. Thus, policymakers should focus on the promising impacts of mindfulness to enhance preschool teachers’ professional identities, beliefs, self-concepts, and positive perceptions or emotions in ECE environments, while also incorporating psychological resilience resources.

## Introduction

Early childhood education (ECE) is an important part of basic education in China, propelling lifelong impacts on children’s physical, mental health, and overall development (Olsen et al., 2024). However, in China and elsewhere, preschool teachers are often and easily subjected to prejudice and bias from society, stemming from lower salaries and social status, limited opportunities for developing professional knowledge, high mobility, and the lack of love for the ECE profession and young children, all of which closely relate to weak professional identity (Hong et al., 2013; Olsen et al., 2024). Based on *China’s Preschool Education Toward 2035*, one key to improving the quality of preschool education is to promote professional identity and professional development of preschool teachers (Jiang et al., 2022; Xiang et al., 2024).

According to Beijaard et al. (2004), the professional identity of teachers as an occupational group is defined as teachers’ sense or perceptions of the role of teaching, relevant features of their profession. During the teacher identification processes, self-concept is dynamic and multifaced (Brickson, 2000; Chen et al., 2023; Jiao et al., 2015). In Brickson’s framework (2000), identification processes contain a set of cognitions about one’s profession, affect reactions toward minority group or organization, and professional behavior. For preschool teachers, forming a professional identity has been highlighted as important in increasing commitment to the profession, perceptions of meaningful work, life satisfaction and well-being (Chen et al., 2023; Olsen et al., 2024). Therefore, it is particularly important to explore multiple forces forming professional identity that might support their more beneficial development. Previous studies have revealed cognitive or psychological factors could be more crucial in motivating teachers’ professional identity (e.g., La Paro, 2015; Xiang et al., 2024).

The broaden-and-build theory described broadened mindsets have adaptive benefits because broadening builds lasting personal resources (Fredrickson and Branigan, 2005). Specifically, the “broaden” means the experience of positive emotions (e.g., interest, joy) may expand one’s awareness and open mindsets. The “broaden” mindsets could build enduring personal resources, such as resilience (Kiken and Fredrickson, 2017), which could support individuals transform themselves and become more identified with themselves (Lutovac et al., 2024). However, to date, social cognitive mindfulness (SCM) as a broadened mindset, is yet unexplored (Langer et al., 2012), particularly in its effect on teachers’ professional identity in the Chinese ECE context. Also unexplored is the potential mediation role of the psychological resource of resilience (Day, 2018) on the SCM and professional identity relationship.

### Mindfulness and preschool teachers’ professional identity

In psychology, Langer’s model of social cognitive mindfulness is defined as a flexible cognitive state in which people actively participate in the present, are sensitive to the environment around them, and notice new things (Langer, 2000; Langer et al., 2012). SCM contains four main subfactors: (1) sensitivity to the environment, (2) the ability to generate new information and create new categories, (3) the ability to think and solve problems from multiple perspectives, and (4) being open to unfamiliar things (Pirson et al., 2012). Previous researchers remain particularly interested in how professionals might develop higher levels of mindfulness (Pirson et al., 2012), since higher levels are associated with personal, interpersonal, and health benefits (Pagnini et al., 2019).

Although SCM has been applied in various fields, the potential effect of mindfulness on preschool teachers’ professional identity remains unknown. However, some clues can be drawn from previous related studies. Prior studies showed that professional beliefs and values were hindered by several psychological or cognitive barriers (Maymin and Langer, 2021), such as professional bias (Hong et al., 2013) and negative attitudes toward work (Xiang et al., 2024). To cope with these cognitive barriers, Moghadam et al. (2022) found mindfulness could be useful to creatively break traditional thinking to utilize a flexible cognitive mindset, wherein teachers are able to make new and novel distinctions. Therefore, SCM could be explored to reimagine and boost attitudes and stances toward work and thus improve preschool teachers’ professional identity.

Previous studies investigating professional identity mainly focus on explicit aspects (Jiao et al., 2015). However, we also hypothesized whether mindfulness could improve teachers’ implicit professional identity. In light of the Dual-Process Theory, individuals’ cognition process consisted of both explicit/conscious/controlled and implicit/unconscious/automatic processing (Payne and Gawronski, 2010). As a dynamic and flexible cognition process in professional practice (Chen et al., 2023), professional identity could be concerned with both implicit and explicit conditions. Based on Greenwald and Banaji (1995), explicit and implicit professional identity are different concepts: the former means attitudes or beliefs that are conscious and deliberately formed; whereas the latter represents the unconscious, automatic, and involuntarily formed attitudes. Notably, mindfulness could positively relate to implicit professional identity. Previous studies strengthened that people being mindful could reduce cognition biases and then boost their decision-making behavior (Langer, 2000; Maymin and Langer, 2021). Hence, in the ECE field, we presumed that SCM could also improve implicit professional identity by reducing some automatic cognition barriers.

The difference between explicit and implicit professional identity is also captured through various measurements. Traditionally, professional identity is measured using self-reported questionnaires (Ding et al., 2022). Since self-reported questionnaires are considered more susceptible to social desirability biases, measurements for testing professional identity implicitly are preferred (Schnabel et al., 2008). The Implicit Association Test (IAT; Greenwald et al., 2009) provides a useful tool to explore individuals’ implicit attitudes or beliefs. Hofmann et al. (2005) work demonstrated that the IAT could measure self-concept, self-esteem, beliefs, and subconscious stereotypes. More importantly, the IAT has been verified to possess predictive validity independent of the predictive validity of explicit measures (Greenwald et al., 2009; Wang et al., 2022). And IAT scores have been shown to be moderately correlated with explicit self-reported questionnaires (Tang et al., 2023). Based on the above literature, the present study explores both explicit professional identities using a self-reported questionnaire and implicit professional identity using IAT.

### The potential mediating role of resilience in the relationship between mindfulness and professional identity

Resilience refers to the capacity to continue “bounce back,” to recover strength or spirit quickly and efficiently in face of adversity (Gu and Day, 2007). The essence of resilience is that some people could cope with stressful experiences better than expected (Parsons et al., 2016). For teachers, they need daily resilience to form and maintain a positive and stable professional identity (Day, 2018). Previous studies showed that a resilient person may have positive coping strategies, such as thinking flexibility, recovery faster and learning more actively strengthen, which could help refine self-identities (Leslie-Miller et al., 2025). As a substantial psychological resource, resilience plays an important role in initiating the motivational process and enhancing professional commitment (Day, 2018; Park et al., 2021). Thus, this paper relies on prior research showing that there could be a connection between resilience and professional identity.

Moreover, previous studies indicate that mindfulness may lead to more active mindsets, freeing limited cognitive resources to respond creatively to situations in a non-habitual fashion (Henriksen et al., 2020). Parsons et al. (2016) found that the capacity to guide automatic cognitive biases is more likely to develop resilience in a wide range of situations. When faced with stress or adversity, one’s flexibility of cognitive processing styles (conscious, goal driven processes) also promoted resilience (Kiken and Fredrickson, 2017; Parsons et al., 2016). In other words, mindfulness might positively predict resilience levels. In addition, Slaymaker et al. (2023) found that resilience plays a mediating role in the relationship between mindfulness and perceived stress or striving. Taken together, we assert that resilience, as an adaptive coping mechanism, could mediate the relationship between SCM and professional identity.

### The current study

The current study aims to investigate the impact of SCM on Chinese preschool teachers’ professional identity and explores the potential mediating role of resilience in this relationship. Combining Langerian mindfulness theory, the broaden-and-build theory, and the Dual-Process Theory, we hypothesized that mindfulness could significantly predict participants’ professional identity, both explicitly and implicitly (Hypothesis 1). In addition, there could be a close relationship established between resilience and professional identity (Hypothesis 2). Resilience mediates the relationship between SCM and professional identity (Hypothesis 3).

## Methods

### Participants

Initially, a total of 110 Chinese female preschool teachers were randomly recruited from preschools in Nanjing, Jiangsu province through online advertisement. Of this sample, two participants who did not meet the criteria for the SC-IAT were excluded from the analysis. One had a higher error rate (error rate > 20%), and the other participant’s latencies of SC-IAT was more than 10,000 ms (Greenwald et al., 2003). Therefore, the final sample was 106 female preschool teachers (mean age = 21.05, *SD* = 2.98). 90 participants (84.9%) were from the public preschool and 16 (15.1%) came from the private preschool. Regarding the years of teaching experience, 55 participants (51.89%) had 5 to 10 years of working as a preschool teacher, 29 (27.4%) had teaching experience below 5 years, and others (22 participants, 20.4%) had more than 10 years teaching experience. In terms of monthly salary, 95 participants (89.6%) earned from 2,200 to 3,000 RMB monthly, and 11 (10.4%) earned above 3,000 RMB every month.

### Measurements

*Langer Mindfulness Scale (LMS).* LMS (Bodner and Langer, 2001) was used in the current study. There are 21 items, such as “I like to investigate things” or “I find it easy to create new and effective ideas.” Each item uses a 7-point Likert-type scale ranging from 1 (strongly disagree) to 7 (strongly agree). A higher score on LMS reflects greater mindfulness level. LMS has been widely used and proved to have high reliability and validity (Geng et al., 2019). In this study, the Cronbach’s *α* of this scale is 0.75. The confirmatory factor analysis indicates a good model fit: *χ^2^/df* = 1.08, comparative fit index (CFI) = 0.91, Tucker–Lewis index (TLI) = 0.90, root mean square error approximation (RMSEA) = 0.027, and standardized root mean residual (SRMR) = 0.08.

*Connor and Davidson’s Resilience Scale (CD-RISC).* To measure preschool teachers’ resilience, the CD-RISC (Connor and Davidson, 2003) with 25 items was adopted. Sample item is “I am confident that I can achieve my goals,” “I can adapt to change,” or “I have a close and secure relationship.” Each item uses a 5-point Likert-type scale ranging from 1 (strongly disagree) to 5 (strongly agree). Higher scores mean preschool teachers possess stronger resilience. The scale has been reported to have good reliability and validity (Bezdjian et al., 2017). In this study, the Cronbach’s *α* is 0.72. The confirmatory factor analysis shows a good fit: *χ^2^/df* = 1.07, CFI = 0.92, TLI = 0.91, RMSEA = 0.025, and SRMR = 0.08.

*Professional Identity Scale (PIS)*. Based on Brickson (2000) and Kremer and Hofman (1985) prior work, Wei (2008) worked to modify and develop the Chinese teachers’ Professional Identity Scale (PIS). Each of the 18 items uses a 5-point Likert-type scale ranging from 1 (strongly disagree) to 5 (strongly agree), such as “I am satisfied with my work as a teacher,” “When the media attack teachers, I feel attacked,” or “I feel comfortable presenting myself as a teacher.” The PIS scale possesses good reliability and strong validity (Ding et al., 2022). In this study, the Cronbach’s α is 0.67, and the confirmatory factor analysis shows a good fit (*χ^2^/df* = 1.06, CFI = 0.91, TLI = 0.90, RMSEA = 0.024, SRMR = 0.07).

*Single-Class Implicit Association Test (SC-IAT).* First, the IAT was developed to explore individuals’ automatic associations between target and attribute words (Greenwald et al., 1998). To measure the implicit professional identity of Chinese preschool teachers, the current study utilized a four-step Single-Class Implicit Association Test (SC-IAT). The SC-IAT is a modification of the original IAT that more accurately measures the strength of the association with evaluation of a single attitude object (Karpinski and Steinman, 2006). Widely used in various fields, the SC-IAT possesses strong reliability and valid psychometric properties (Tang et al., 2023). The SC-IAT consists of 192 items in four sections or blocks (24 trials in two practicing blocks and 72 trials in two testing blocks). All SC-IAT trials were counterbalanced, with words/concepts in the stems randomly ordered. Participants were asked to complete a categorization task: one distinguishing between words suggesting “positive profession perception” (e.g., lucky, delightful, happy, cheerful and joyful) and “negative profession perception” (e.g., sick, cruel, horrible, miserable, and disgusting); the other representing words suggesting “preschool teachers’ work with curriculum contents,” such as the creating a learning environment, picture books, the piano, dancing, and preschool-home collaboration. Then, participants were instructed online to respond to two kinds of pairings by clicking different keys according to instructions: One pair was compatible (e.g., “positive profession perception” and “curriculum contents”) and the other was incompatible (e.g., “negative profession perception” and “curriculum contents”). Based on Greenwald et al. (2003) prior work, the more implicit associations one experiences, the easier and faster he/she would categorize a compatible pairing. The final indicator of the SC-IAT is D-score (−2 to 2), which is the difference between the mean response time for compatible trials and the mean response time for incompatible trials (Greenwald et al., 2009). That is, larger D-scores indicate stronger implicit professional identity of preschool teachers.

*Control* Var*iables.* Prior research also found that professional identity may be influenced by certain demographic characteristics (Olsen et al., 2024), such as age and whether preschool is public or private, years of teaching experience and monthly salary. In the current study, these variables were controlled as covariates during data analysis.

### Procedure

The whole research was implemented in accordance with guidelines by the Ethics Committee of Nanjing Xiaozhuang University. All assessments were presented in Chinese. First, participants were tested individually in private rooms after work. Then, after signing the informed consent form, they completed the LMS, CD-RISC, SC-IAT and PIS scales through the computer. Finally, they were thanked and debriefed. The whole investigation lasted about 15 min.

### Data analysis

The data were analyzed using SPSS 26.0 and Amos 23.0. Specifically, (1) we used Amos 23.0 to perform the confirmatory factor analysis and test the construct validities of main variables; (2) SPSS 26.0 was utilized to examine Harman’s single-factor test; (3) descriptive statistics and correlations were performed; (4) Amos 23.0 was used to control covariates and explore the relationship mechanism among SCM, resilience, explicit and implicit professional identity.

## Results

### Common method bias

Harman’s single-factor test was used to check for common method bias (Podsakoff et al., 2003). There were 24 factors with eigenvalues greater than 1, and the first factor explained 10.25% of the variance, showing common method bias was not a significant concern.

### Descriptive analyses and correlations

Descriptive analyses and correlations were performed using SPSS 26.0. As shown in Table 1, skewness and kurtosis values are within the critical criterion (skewness < |2.0|, kurtosis < |7.0|, Hancock et al., 2018), which showed variables were normally distributed. Besides, all variables are highly related.

**Table 1:** Means, standard deviation, and correlations for the main variables.

Variables	1	2	3	4
1. SCM	1			
2. Resilience	0.48^**^	1		
3. Professional identity	0.41^**^	0.48^**^	1	
4. *D*-score	0.58^**^	0.53^**^	0.25^*^	1
*M*	88.90	77.69	55.89	0.83
*SD*	13.47	9.80	8.87	0.15
Skewness	0.44	0.40	0.91	−0.66
Kurtosis	0.39	−0.28	0.18	1.18

*N =* 106. SCM, social cognition mindfulness; D-score is the indicator of the Single-Class Implicit Association Test (the same below). ^**^p < 0.01; ^*^p < 0.05.

### Mediation test: explicit and implicit professional identity

To explore whether resilience might mediate the effect of SCM on professional identity, the present study built a structural equation modeling (SEM) using Amos 23.0. Specifically, the LMS score served as the independent variable, the CD-RISC score served as the mediator variable, the explicit professional identity and the SC-IAT *D*-score served as the dependent variables; with age and private/public preschool, years of teaching experience, and monthly salary controlled as covariates. This model showed a good fit to the empirical data. All the indices fulfilled the criteria: *χ^2^ /df* = 1.35, CFI = 0.97, TLI = 0.94, RMSEA = 0.057, SRMR = 0.06.

**Figure 1:**
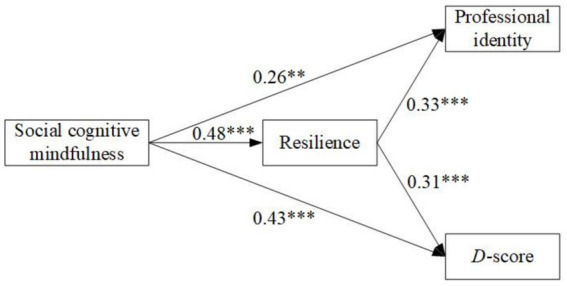
Mediation test: explicit and implicit professional identity. *N* = 106. This figure presents the mediating role of resilience in the relationship between SCM and explicit and implicit professional identity, respectively. Results are standardized coefficients. **p < 0.01, ***p < 0.001.

Figure 1 illustrates the total effects of SCM on resilience is 0.48 (*p* < 0.01, SE = 0.09, 95% CI [0.26, 0.65]), supporting our second hypothesis. The total effects of SCM on explicit and implicit professional identity were 0.41 (*p* < 0.001, SE = 0.10, 95% CI [0.18, 0.58]) and 0.58 (*p* < 0.01, SE = 0.08, 95% CI [0.44, 0.68]), respectively. The direct effects of SCM on explicit and implicit professional identity were 0.26 (*p* < 0.01, SE = 0.13, 95% CI [0.03, 0.48]) and 0.43 (*p* < 0.001, SE = 0.09, 95% CI [0.23, 0.58]), respectively. The indirect effects of SCM on explicit and implicit professional identity were 0.16 (*p* < 0.01, SE = 0.07, 95% CI [0.05, 0.32]) and 0.15 (*p* < 0.001, SE = 0.07, 95% CI [0.04, 0.31]), respectively. Results confirm Hypothesis 3 that resilience mediates the relationship between SCM and professional identity, explicitly and implicitly.

## Discussion

The current study tested the effect of SCM on increasing both explicit and implicit professional identity and examined the indirect impact of resilience on the relationship between SCM and professional identity. Our first hypothesis that SCM could directly and significantly promote preschool teachers’ professional identity both explicitly and implicitly, was supported. Resilience positively linked with professional identity, which supported our second hypothesis. The mediation test also proved the third hypothesis that SCM indirectly influences preschool teachers’ professional identity through resilience.

### Mindfulness influences professional identity for preschool teachers

Results confirmed the positive direct impact of SCM on professional identity among preschool teachers, which broadens the scope of SCM research in ECE contexts. Being mindful indicates individuals are aware of varying contexts, welcome what is new, and create novel ways of thinking by using new categories for structuring perceptions (Langer et al., 2012). Hence, preschool teachers who were identified as mindful also possessed a flexible mindset that could draw distinctions about professional processes, provide various answers to each question, and inspire multi-perspective thinking, leading to a more positive self or role in their daily professional lives.

Notably, our findings also extend the association of SCM to explicit and implicit professional identity and verify the Dual-Process Theory (Payne and Gawronski, 2010). Consistent with Weber (2017) work, individuals usually prefer automatically activated associations rather than rational analysis to guide their attitudes and behaviors, which reflects unconsciousness. The unconscious cognition may be the implicit bias on stressed-related prolonged activity, but could be reduced by mindfulness (Brosschota et al., 2010). According to Langerian mindfulness theory (Maymin and Langer, 2021), one of the powers of mindfulness is decreasing implicit bias in the workplace. It is easier for mindful preschool teachers to be aware of and control the implicit bias or automatic reaction triggered by stressful events (Langer, 2000). Being mindful means that they have more open mind thinking mode and cognitively activate to engaging in conscious and automatic processes. Combined, SCM could enhance preschool teachers’ professional identity, both explicitly and implicitly.

### The mediating role of resilience

In addition to the direct effect of SCM on preschool teachers’ professional identity, the present study also explored an indirect effect, in which resilience plays a mediating role. Consistent with prior studies (Kiken and Fredrickson, 2017; Leslie-Miller et al., 2025), when confronted with stress, setbacks, and difficulties during work, resilient preschool teachers typically reported more mindfulness-related coping skills to generate many, varied solutions. Resilience as a mediating role also aligns with Identity Process Theory (IPT) wherein resilience acts as a recovery of functioning, while positive professional identity emphasizes a higher level of functions to handle challenges and changes related to work tasks (Breakwell et al., 2021). In other words, resilient preschool teachers could integrate challenging experiences into stronger self-concept and act in ways that are authentic to their values, thereby solidifying a positive and durable professional self.

Moreover, the current study supports that SCM was associated with increased resilience. When participants are in a broaden state of mindfulness, they simultaneously produced higher levels of resilience despite uncertainty or adversity (Pagnini et al., 2019; Slaymaker et al., 2023). According to the broaden-and-build theory, psychological resilience is likely a finite resource that needs to be replenished; therefore, mindfulness is a key strategy to build and maintain resilience (Fredrickson and Branigan, 2005; Lutovac et al., 2024). In Langer’s social-cognitive perspective, mindfulness leverages novelty-seeking to garner and support cognitive flexibility, which improves resilience skills. For preschool teachers, being in a state of mindfulness could break the stressful routine when difficulties are encountered and allow exploration of various adaptive coping abilities (e.g., flexible thinking, emotion control, psychical exercises, Leslie-Miller et al., 2025). Taken together, SCM could also boost positive professional identity through the mediation effect of resilience.

### Implications

To date, our study is the first to apply SCM to enhance the professional identity of preschool teachers and successfully test the mediating role of resilience. Considering the innovation value of the present study, practical and theoretical implications are recommended in the ECE field. In the theoretical research domain, many new creative models are based on the SCM theory. For example, the mindfulness-cultivation technique was developed to predict people’s reflective thinking and positive orientation (Moghadam et al., 2022). Existing applications of SCM somewhat supported that mindfulness could influence positive feelings or senses of being a preschool teacher and not only reduce negative identity crisis as shown in earlier studies. Therefore, policymakers and education bureaus should make efforts to provide positive, innovative ECE information in multiple ways (e.g., mass media, networks, advertising for good working atmosphere, improvements in professional status, salaries, and working conditions), which could continually broaden preschool teachers’ mindsets to strengthen early childhood professional identity.

Second, mindfulness could also be critical for improvement in resilience and recovery in general population (Pagnini et al., 2019), which can further foster positive professional belief and behaviors. That is, considering the significant mediation role of resilience on the relationship between SCM and professional identity, it is promising to increase the resilience level of preschool teachers by using novel resiliency measures. For example, using principles from Acceptance and Commitment Therapy (ACT; Aravind et al., 2024) to help preschool teachers learn how to accept difficult thoughts and feelings without being controlled by them, and to still act aligned with their teaching values and beliefs. Park et al. (2021) employed the Resiliency Treatment Model (RTM) to describe stress management, resiliency training or relaxation response resiliency programs should be developed to explore professional adaptive strategies. In China, normal universities [shifan daxue] may consider using the ACT or RTM to improve preservice and in-service training systems by increasing positive psychological states, social connectedness, positive affect, empathy, intrinsic motivation, and job satisfaction for prospective and current early childhood teachers.

### Limitations and future directions

The current study has several limitations. First, sampling bias may exist because of the geographic boundary of the province. Extending the current study to larger and/or broader demographics, such as male preschool teachers, primary or middle school teachers, and across cultural backgrounds, will make an even greater contribution to the field. Meanwhile, for conducting Structural Equation Modeling (SEM), it typically requires larger samples to achieve more stable parameter estimates and adequate statistical power. Further research could also utilize alternative methods for goodness-of-fit evaluation and reduce the potential measurement error with increasing sufficient sample sizes (Xia and Yang, 2019). Then, this is a cross-sectional study, which may generate substantially biased estimates of longitudinal parameters even under ideal conditions when mediation is complete (Maxwell and Cole, 2007). Therefore, multiple research methods (i.e., interventional studies, behavioral markers and observations; Large Language Models, LLM) could focus on the possible long-term impact of SCM on professional identity. For example, future longitudinal or experimental intervention studies could help to test these causal pathways more rigorously. Finally, apart from the direct effects, the mediation investigation also confirmed a significant indirect effect of SCM on professional identity, indicating the potential for other variables to play a possible mediating or moderating role (Slaymaker et al., 2023). Particularly, more empirical evidence on how SCM promotes preschool teachers’ professional identity (e.g., through autonomy, structure, emotional regulation) is needed.

Despite being an initial probe into SCM, our research could serve as an exploratory and anchoring study in the ECE field, lending preliminary support to the claim that SCM could significantly and positively influence preschool teachers’ professional identity. It not only focuses on professional identity but also suggests SCM could contribute to promoting job satisfaction, self-esteem, perceived ability, and employee engagement in the workplace (Slaymaker et al., 2023; Xiang et al., 2024). More importantly, by examining the mediating role of resilience as a powerful mechanism across mindfulness, as well as explicit and implicit professional identity, this study sheds light on innovative, practical, relatively expedient and inexpensive ways to advocate for preschool teachers’ high quality professional development and well-being. No doubt, future research should also focus on enacting and broadening the impacts of SCM for teachers in early-childhood educational contexts.

## Data Availability

The raw data supporting the conclusions of this article will be made available by the authors, without undue reservation.
